# Comparison of Noninvasive Methods for the Evaluation of Liver Fibrosis in Children With Chronic Hepatitis C Virus Infection

**DOI:** 10.1097/INF.0000000000004978

**Published:** 2025-09-11

**Authors:** Maria Pokorska-Śpiewak, Anna Dobrzeniecka, Ewa Talarek, Małgorzata Aniszewska, Magdalena Marczyńska

**Affiliations:** From the * Department of Children’s Infectious Diseases, Medical University of Warsaw, Warsaw, Poland; † Department of Pediatric Infectious Diseases, Regional Hospital of Infectious Diseases in Warsaw, Warsaw, Poland.

**Keywords:** hepatitis C virus, elastography, aspartate transaminase-to-platelet ratio index, fibrosis-4 index, liver fibrosis

## Abstract

**Background and Aims:**

This study aimed to analyze liver fibrosis using transient elastography (TE) and serum biomarkers [aspartate transaminase-to-platelet ratio index (APRI) and the fibrosis-4 index (FIB-4)] in children with chronic hepatitis C before antiviral treatment and to compare the results of these noninvasive methods.

**Methods:**

All consecutive patients 3–17 years old treated with direct-acting antivirals for hepatitis C virus infection between August 2019 and July 2024 were included. Evaluation of liver stiffness measurement (LSM) was performed before starting treatment with TE. Liver fibrosis was considered significant if the median LSM was >7 kPa, corresponding to a METAVIR F score of ≥2 points. Simultaneously, TE, APRI and FIB-4 evaluations were performed, and their accuracy in the detection of significant fibrosis and cirrhosis was determined by calculating areas under the receiver operating characteristic curve (AUROC) using the LSM results as a reference.

**Results:**

One hundred fifty patients with a median age of 11 years were included. TE evaluation revealed that 139/150 (92.7%) of the participants presented with normal LSMs (≤7.0 kPa), whereas in the remaining 11/150 (7.3%) participants, significant fibrosis was confirmed, correlating to a score of F2 on the METAVIR scale in 6 (4%), F3 in 2 (1.3%) and F4 in 3 (2%). Among the independent predictors of significant fibrosis were age >10 years and duration of infection >10 years. The median APRI and FIB-4 values were significantly greater in children with significant liver fibrosis on TE evaluation. For detecting significant fibrosis, the AUROC was 0.706 for the APRI and 0.802 for the FIB-4, with cutoff values >0.53 for the APRI and >0.24 for the FIB-4. When the accuracies of the APRI and the FIB-4 for detecting cirrhosis were analyzed, the AUROCs were greater: 0.879 for the APRI, with a cutoff >0.53, and 0.96 for the FIB-4, with a cutoff >0.40.

**Conclusion:**

There is some agreement between the results of biomarker (APRI and FIB-4) and TE evaluation, but with the assumption of lower cutoff thresholds indicating significant fibrosis/cirrhosis than previously validated in adults.

It has been estimated that more than 3 million children are infected with hepatitis C virus (HCV) globally.^1^ Although up to 40% of these patients may spontaneously clear the HCV during the first 4–7 years of life, the majority develop chronic hepatitis C (CHC), which is a progressive disease.^2^ CHC may result in liver fibrosis, which can lead to cirrhosis, decompensated liver disease and hepatocellular carcinoma. According to recent British data, based on approximately 1500 patients observed longitudinally, liver cirrhosis may occur in one-third of patients infected with HCV during childhood, over a median of 33 years after infection.^3^ Our preliminary observations of 35 teenagers 12–17 years old revealed that more than 10% of adolescents with CHC may present with significant liver fibrosis.^4^

In recent years, noninvasive methods have replaced liver biopsy for the evaluation of liver fibrosis in patients with CHC, and they have improved the diagnosis and prognostication of chronic liver diseases.^5^ Among them, transient elastography (TE) and serum biomarkers are most widely used, followed by shear wave elastography and magnetic resonance elastography.^5^ Available data suggest that both TE and serum markers, such as the aspartate transaminase-to-platelet ratio index (APRI) and fibrosis-4 (FIB-4) index, accurately discriminate between patients with and without significant fibrosis.^6,7^ However, most of these methods have not been satisfactorily validated in pediatric patients. In children, liver fibrosis is usually less common than it is in adult patients; thus, the utility of these tests may be limited. In this study, we aimed to analyze liver fibrosis using TE and serum biomarkers (APRI and FIB-4) in children with CHC before antiviral treatment and to compare the results of these noninvasive methods.

## METHODS

### Patients

In this study, we included all consecutive patients 3–17 years of age who were treated with direct-acting antivirals (DAAs) for HCV infection in our tertiary health care department between August 2019 and July 2024. Children in Poland have not been included in the national therapeutic programs for CHC. However, in our department, we have led 2 projects that enabled treatment for pediatric patients infected with HCV: the PANDAA-PED study (“Treatment of chronic hepatitis C in children 6–18 years of age using a pangenotypic direct-acting antiviral sofosbuvir/velpatasvir”) and the POLAC project. In brief, the PANDAA-PED study^8^ was a noncommercial, nonrandomized, open-label study funded by the Medical Research Agency, Warsaw, Poland (grant number 2019/ABM/01/00014). In this project, 50 patients 6–18 years old were successfully treated for HCV infection between January 2022 and October 2022 using a 12-week course of sofosbuvir/velpatasvir (SOF/VEL) fixed-dose adjusted to body weight.^8^ The POLAC Project is a real-life therapeutic program (“Treatment of Polish Children and adolescents with chronic hepatitis C using direct acting antivirals”) launched in our department for HCV-infected children in August 2019, first for children at least 12 years of age and then (in April 2021) extended for patients 3 years old and above from all Polish regions.^9,10^ The program is available courtesy of the donation of DAAs [sofosbuvir/ledipasvir (SOF/LDV) and glecaprevir/pibrentasvir (GLE/PIB)] by pharmaceutical companies.

In both projects, a similar protocol was used. Children were qualified for treatment in the case of confirmed CHC based on the positive HCV polymerase chain reaction RNA testing for at least 6 months, irrespective of the HCV genotype (for SOF/VEL and GLE/PIB treatment), or infected with HCV genotypes 1 or 4 in the case of SOF/LDV therapy. The primary endpoint for these studies included evaluation of DAA efficacy (defined as sustained virologic response 12, with undetectable HCV RNA at 12 weeks posttreatment).^8–10^

### Evaluation of Liver Fibrosis and Steatosis

Evaluation of liver fibrosis [liver stiffness measurement (LSM)] was performed at baseline (before starting treatment) by TE by certified trained examiners using a FibroScan device (Echosens, Paris, France). The final LSM result was the median value of at least 10 valid measurements. It corresponded to liver fibrosis on the METAVIR scale according to the Castera TE cutoffs as follows: no to mild fibrosis (F0/1), LSM up to 7.0 kPa; moderate fibrosis (F2), LSM 7.1–9.4 kPa; severe fibrosis (F3), LSM 9.5–12.4 kPa; and cirrhosis (F4), LSM ≥12.5 kPa.^11^ Liver fibrosis was considered significant if the median LSM was >7 kPa, corresponding to a METAVIR F score of ≥2 points.

In addition to TE, a biomarker evaluation was performed, which included 2 indirect fibrosis biomarkers, the APRI and FIB-4, which were calculated according to the analytic recommendations^6,7^:

APRI = [(AST (IU/L)/AST ULN)/platelet count (10^9^/L)] × 100

FIB−4 = [Age (years) × AST (IU/L)]/[platelet count (10^9^/L) × √ALT (IU/L)].

The following cutoffs were considered to suggest significant fibrosis: APRI >0.7 and FIB-4 >1.45.^6,12^ For this study, TE was considered a reference method for liver fibrosis measurement.

### Data Collection

Demographic, epidemiologic, clinical and virologic data were collected and analyzed. The most likely mode of HCV infection was established using available medical records. At baseline, all participants underwent physical examination and laboratory testing, including blood cell count, alanine aminotransferase, and aspartate aminotransaminase levels (ALT and AST), total bilirubin, albumin, creatinine concentrations and prothrombin index evaluation. Biochemical serum testing was performed using commercially available laboratory kits. For both ALT and AST, upper limits of normal were set at 40 IU/mL. HCV RNA determination was performed using quantitative real-time polymerase chain reaction (Test Cobas 5800 HCV, Roche Diagnostics, Rotkreuz, Switzerland; with measurement linearity range of 15–1.0 × 10^8^ IU/mL). Body mass index (BMI) z-scores were calculated using the World Health Organization (WHO) Child Growth Standards and Growth Reference data with the WHO anthropometric calculator, AnthroPlus v.1.0.4. All patients were screened for hepatitis B virus (HBV) and HIV coinfection based on evaluation of HBs antigen and anti-HBc antibodies, or anti-HIV antibodies, respectively.

### Statistical Analysis

Data are presented as numbers (percentages of total) or medians (interquartile ranges, IQRs), as appropriate. Categorical variables were compared using a χ^2^ test, and continuous data were compared using the Mann–Whitney test. To analyze correlations between different noninvasive biomarkers, Spearman’s rho correlation coefficients with 95% confidence intervals (CIs) were calculated.

To identify predictors of liver fibrosis, a linear regression analysis was conducted. Stepwise multivariate logistic regression was used to determine factors associated with liver fibrosis (F≥2 on METAVIR scale). The following variables were entered into the model irrespective of the results of the univariate analysis: age, duration of infection, HCV genotype, sex, ALT, AST, total bilirubin, platelets, BMI, BMI z-score and HCV viral load. Considering a strong correlation between patients’ age and disease duration, 2 separate models were constructed to avoid multicollinearity: model 1, including age, and model 2, including duration of HCV infection.

The diagnostic performance of the APRI and FIB-4 for identifying patients with significant fibrosis or cirrhosis was assessed by calculating receiver operating characteristic (ROC) curves and areas under the ROC curve (AUROC) with the LSM results as a reference. An analyzed test was considered perfect when the AUROC was 1.0, excellent when the AUROC was over 0.9, and good when the AUROC was over 0.8. A two-sided *P* value of < 0.05 was considered significant. Statistical analyses were performed using MedCalc Statistical Software version 22.018 (MedCalc, Ostend, Belgium).

## RESULTS

### Participants

We included 150 patients who were eligible for treatment with SOF/LDV (40 children), SOF/VEL (50 children) or GLE/PIB (60 children). The baseline epidemiologic and laboratory characteristics of the study group are presented in Table 1. The median age of the participants was 11 years, with a range of 3–17 years. Most children were infected with genotype 1 HCV, and the majority had become infected vertically from their mothers. Two patients were coinfected with HIV, and 2 were coinfected with inactive HBV.

**TABLE 1.** Baseline Characteristics of the Study Group According to the Liver Stiffness Measurement

Parameter	Whole Group (n = 150)	Patients with LSM Corresponding to F0/1* (n = 139)	Patients with LSM Corresponding to F ≥2* (n = 11)	*P* Value
Age (years)	11 (7–13)	11 (7–13)	13 (12–15)	0.007
Sex (male)	79 (53%)	72 (52%)	7 (64%)	0.45
HCV genotype				
1	113 (75%)	105 (76%)	8 (73%)	0.84
3 or 4	37 (25%)	34 (24%)	3 (27%)	
Vertical mode of infection	135 (95%)	125 (90%)	10 (91%)	0.91
Previous ineffective treatment with IFN+RBV	19 (13%)	14 (10%)	5 (45%)	0.0007
Coinfection with HIV	2 (1%)	1 (1%)	1 (9%)	0.02
Coinfection with HBV	2 (1%)	1 (1%)	0	0.68
Duration of infection†	10 (7–13)	10 (7–13)	13.5 (12–15)	0.002
BMI	18.0 (15.5–21.1)	17.7 (15.5–20.7)	21.0 (20.3–25.4)	0.004
BMI z-score	0.2 (−0.5 to 1.0)	0.1 (−0.5 to 0.96)	0.7 (0.5–1.6)	0.03
ALT (IU/mL)	45 (33–65)	45 (33–64)	52 (34–108)	0.25
AST (IU/mL)	48 (36–61)	47 (35–59)	62 (30–81)	0.49
Total bilirubin (µmol/L)	10.1 (7.4–13.6)	10.1 (7.4–13.5)	11.8 (8.3–16.9)	0.38
Platelets (G/L)	305 (252–353)	313 (261–356)	221 (195–281)	0.001
Hemoglobin (g/dL)	13.5 (12.7–14.3)	13.5 (12.6–14.1)	14.3 (13.8–15.1)	0.006
Albumin (G/L)	46.6 (45.0–48.5)	46.7 (45.0–48.5)	45.0 (42.3–47.1)	0.23
Creatinine (µmol/L)	45 (38–53)	45 (38–52)	60 (45–66)	0.01
Prothrombine index (%)	96 (91–100)	96 (91–100)	100 (94–105)	0.07
HCV viral load (log_10_ IU/mL)	5.8 (5.3–6.3)	5.8 (5.3–6.3)	5.6 (5.1–6.3)	0.79
LSM (kPa)	4.5 (3.7–5.4)	4.4 (3.7–5.2)	8.9 (7.6–12.2)	<0.0001
CAP (dB/m)	188 (160–209)	185 (158–209)	209 (193–248)	0.004
APRI	0.37 (0.28–0.51)	0.36 (0.28–0.50)	0.55 (0.37–0.64)	0.02
FIB-4	0.24 (0.16–0.34)	0.23 (0.16–0.32)	0.36 (0.27–0.52)	0.0009

* on METAVIR scale.

† Estimated for 137 (127 + 10) children.

CAP indicates controlled attenuation parameter; IFN, interferon; RBV, ribavirin.

Data are presented as medians (interquartile ranges) or numbers (percentages), as appropriate.

### Evaluation of Liver Fibrosis by TE

TE evaluation revealed that 139/150 (92.7%) of the participants presented with normal liver stiffness (LSM ≤7.0 kPa), whereas in the remaining 11/150 (7.3%), significant fibrosis was confirmed, correlating to a score of F2 on the METAVIR scale in 6 (4%), F3 in 2 (1.3%) and F4 in 3 (2%).

### Predictors of Liver Fibrosis on TE Evaluation

A comparison of epidemiologic and laboratory features in children according to the extent of liver fibrosis in TE evaluation is presented in Table 1. Compared with those with normal liver stiffness, children with significant fibrosis were older and had a longer duration of HCV infection. In addition, these patients more frequently received previous ineffective treatment with interferon and ribavirin, and they were more often coinfected with HIV. Children who presented with significant fibrosis also had higher median BMIs and BMI z-scores. The median platelet count was lower in these patients. In addition, their hemoglobin level was greater, and their creatinine concentration was lower, but these observations were probably related to the older age of these patients.

**FIGURE 1.**
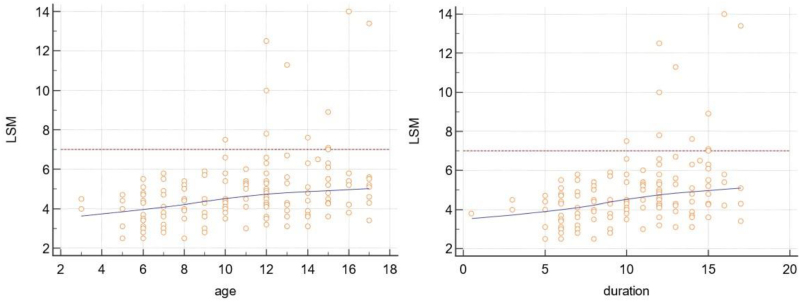
Scatter diagrams showing liver stiffness measurements (LSMs) according to patient age and duration of infection in the study group. The blue lines represent trend lines, and the red lines represent the reference lines corresponding to the LSM of 7.0 kPa.

Logistic regression analysis revealed that among the independent predictors of significant fibrosis were age >10 years, duration of infection >10 years, higher BMI value, higher AST level, and lower platelet count (Table 2). Interestingly, as shown in Figure 1A, only children at least 10 years of age or with at least a 10-year duration of HCV infection presented with significant liver fibrosis. When we performed a multivariate analysis, in both models (with patients’ age and duration of HCV infection), only lower platelet count was found to be an independent predictor of liver fibrosis (odds ratio: 0.98, 95% CI: 0.97–0.99).

**TABLE 2.** Predictors of Liver Stiffness Measurement Corresponding to F≥2 on the METAVIR Scale (Univariate Logistic Regression Analysis)

Predictor	OR (95% CI)	*P* value
Age >10 years	9.57 (1.2–76.8)	0.005
Duration of HCV infection >10 years	10.37 (1.27–84.30)	0.004
Genotype 1 vs. other	3.20 (0.29–34.35)	0.26
Sex (male vs. female)	1.58 (0.44–5.64)	0.47
ALT	1.0 (0.99–1.01)	0.15
AST	1.01 (1.01–1.03)	0.04
Total bilirubin	1.01 (0.95–1.08)	0.60
Platelets	0.98 (0.97–0.99)	0.0002
BMI	1.18 (1.05–1.32)	0.005
BMI z-score	1.64 (0.98–2.73)	0.05
HCV viral load	1.04 (0.48–2.25)	0.80

OR, odds ratio.

### Comparison Between TE and Biomarker Evaluation

As shown in Table 1, both the APRI and FIB-4 median values were significantly greater in children with significant liver fibrosis on TE evaluation than in those with normal liver stiffness. In addition, there was a correlation between the LSM and both the APRI and FIB-4 values (Table 3).

**TABLE 3.** Correlation Between Liver Stiffness Measurements and Serum Biomarkers

Markers	r (95% CI)	*P* value
APRI vs. LSM	0.35 (0.20–0.48)	<0.0001
FIB-4 vs. LSM	0.50 (0.37–0.61)	<0.0001
APRI vs. FIB-4	0.68 (0.58–0.76)	<0.0001

r indicates correlation coefficient.

**FIGURE 2.**
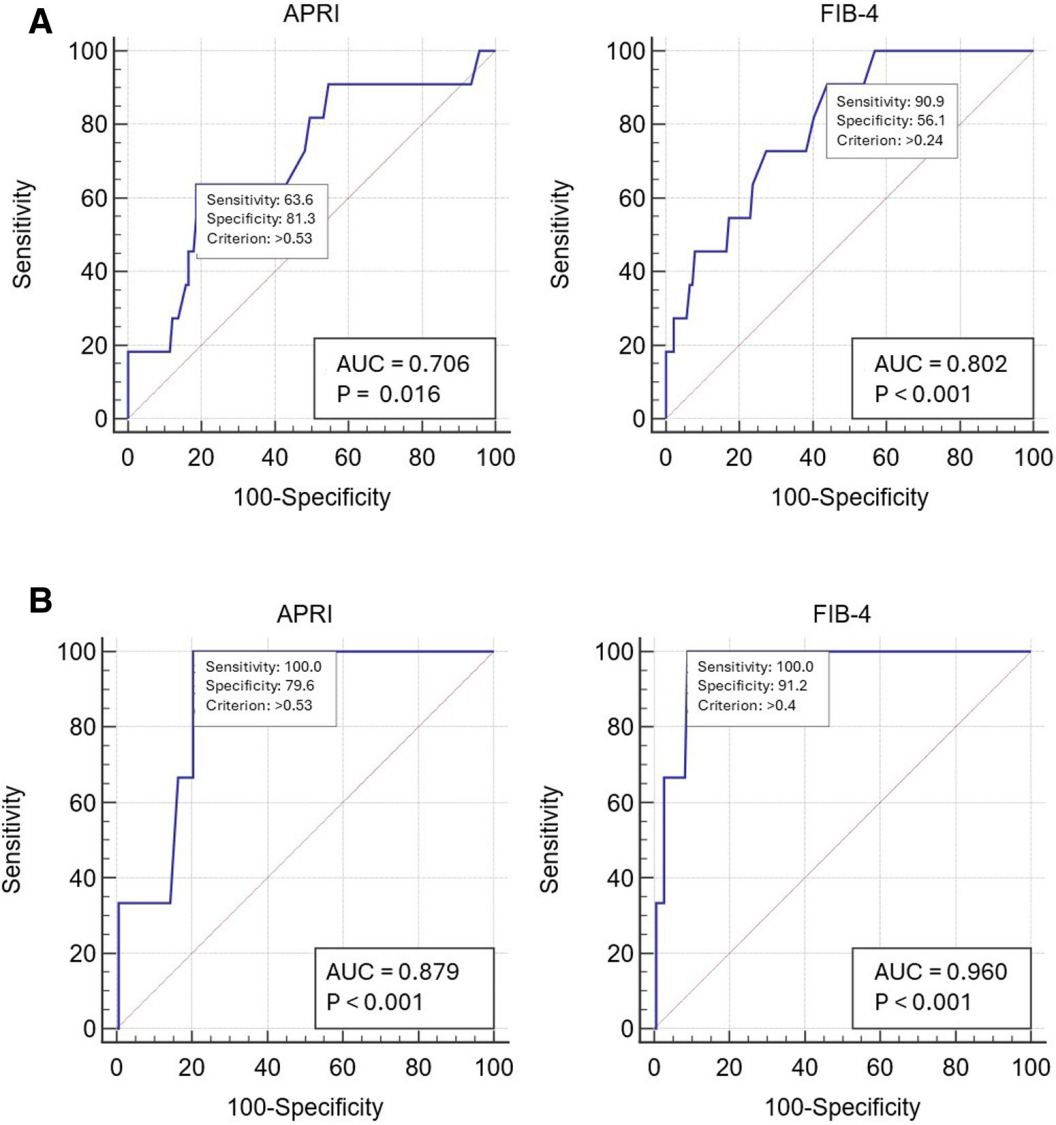
Receiver operating characteristic (ROC) curves of the APRI and FIB-4 for detecting (A) significant liver stiffness (LSM >7 kPa, METAVIR F≥2) in transient elastography evaluation; (B) cirrhosis (LSM>12.5 kPa, METAVIR F4) in transient elastography evaluation.

When we analyzed the accuracy of the APRI and FIB-4 for detecting significant fibrosis during TE evaluation (Fig. 2A), we found that the AUROC was 0.706 for the APRI and 0.802 for the FIB-4. However, the suggested cutoff thresholds (>0.53 for the APRI and >0.24 for the FIB-4) were significantly lower than those suggested for significant fibrosis in adults (>0.7 and >1.45 for the APRI and FIB-4, respectively). When the accuracies of the APRI and FIB-4 for detecting cirrhosis were analyzed (Fig. 2B), the AUROCs were greater: 0.879 for the APRI, with a cutoff >0.53, and 0.96 for the FIB-4, with a cutoff >0.40.

**FIGURE 3.**
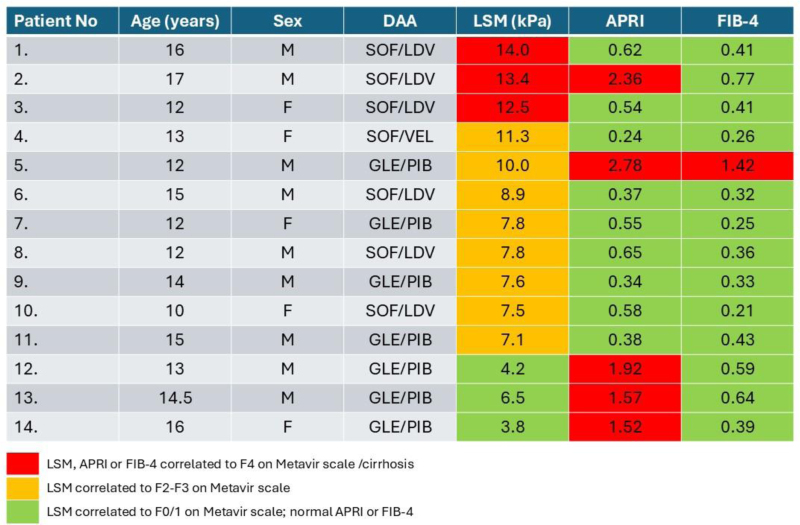
A comparison of the results of transient elastography and biomarker evaluation, presenting all patients for whom at least one method revealed significant fibrosis (using cutoff values for the APRI and FIB-4 validated previously for adults, >0.7 and >1.45, respectively). F indicates female; M, male.

In Figure 3, we present a comparison of the results of TE and biomarker evaluation, showing all patients in whom at least 1 method revealed significant fibrosis (using cutoffs for the APRI and FIB-4 validated previously for adults, >0.7 and >1.45, respectively). This analysis revealed that the use of these cutoff thresholds for biomarkers leads to several discrepancies in the assessment of liver fibrosis in children with CHC.

## DISCUSSION

In this study, we demonstrated that 7.3% of children 3–17 years old with CHC may develop significant liver fibrosis, including 2% presenting with cirrhosis. Age and duration of HCV infection over 10 years were among the predictors of significant fibrosis. This observation should be interpreted with caution, as the participants were examined with respect to their qualifications for DAA treatment, which, before April 2021, was available only for children at least 12 years of age. Thus, it is possible that significant fibrosis might have developed earlier during childhood. In a study by Turkova et al^13^ on a large cohort of pediatric patients with CHC, 8 (4%) of 223 patients evaluated with TE presented with significant fibrosis, and an association between older age and increased LSM was found. In this study, no child younger than 7.5 years old presented with significant fibrosis.^13^ This confirms the results of previous studies, where, according to histopathologic evaluation, older age and longer duration of CHC were associated with a greater risk of disease progression.^14,15^ All these data suggest that although progression to advanced liver disease is not common in children with CHC, it is still possible, as several studies have shown the possibility of progression to cirrhosis during childhood.^4,16–18^ In addition, we found that lower platelet count is an independent predictor of liver fibrosis in children with CHC. It has been shown that a decreased platelet count can indicate liver fibrosis, as particularly advanced fibrosis reduces platelet counts by reducing thrombopoietin generation by hepatocytes and hypersplenism.^19^ As the factors associated with liver disease progression in children with HCV infection are still not clear, it is impossible to predict who would develop disease complications such as cirrhosis, portal hypertension or hepatocellular carcinoma and thus who should receive prompt treatment.^20^ In the era of safe and extremely effective DAA therapies, it seems reasonable to treat all pediatric patients at least 3 years of age, as soon as possible, to avoid these complications.

The main purpose of this study was to compare TE and biomarker evaluations to analyze the accuracy of the APRI and FIB-4 for diagnosing significant fibrosis in children with CHC. We found that both markers may be accurate in detecting cirrhosis and, to a lesser extent, significant fibrosis, but only when lower cutoff thresholds are used. Noninvasive tests have satisfactorily replaced liver biopsy for the evaluation of liver fibrosis, and they are now the method of choice for prognostication in patients with chronic viral hepatitis.^5^ Liver biopsy is an invasive procedure that may rarely lead to severe complications.^4^ Thus, noninvasive, repeatable and, if possible, less expensive alternatives for the assessment of liver fibrosis are highly desirable. Among them, TE is the most widely used and validated point-of-care technique with good reproducibility and high performance for cirrhosis.^5^ However, it requires a dedicated expensive device. Thus, in settings not equipped with TE devices, serum biomarkers such as the APRI or FIB-4 might be useful alternatives. They have good reproducibility, low cost and high applicability, and they have been validated for some etiologies of liver diseases in adult patients.^5,21,22^

In this study, we used TE as a reference method, as according to some previous papers, TE evaluation was positively correlated with histopathologic evaluation.^23,24^ There is also some evidence in children with chronic hepatitis B that TE is a superior noninvasive index to the APRI for detecting liver fibrosis.^25–27^ ElShahawy et al^28^, who analyzed the accuracy of noninvasive methods (TE, APRI and hyaluronic acid) for the diagnosis of liver fibrosis in 50 children with chronic HCV and HBV infection compared with histopathologic evaluation, reported that the accuracy of TE and APRI compared with liver biopsy was 64% for both methods, with a confidence interval of 95%. Compared with those of histopathologic evaluation, the sensitivities of TE and APRI were 60.5% and 65.1%, whereas their specificities were 85.7% and 57.1%, respectively. The authors concluded that TE and the APRI are both good indicators of liver fibrosis in children with almost the same accuracy; however, TE was the only method that enabled differentiation between mild and significant fibrosis.^28^ Notably, our study revealed significantly higher AUROCs for both the APRI and FIB-4 for the detection of significant fibrosis and cirrhosis.

Our study provides new data on lower cutoff thresholds for the APRI and FIB-4 for the detection of significant fibrosis and cirrhosis in a large cohort of children with CHC. However, several limitations of this study should be considered. First, we did not perform liver biopsy, and we used the results of TE evaluation as a reference method instead of histopathologic analysis. Currently, liver biopsy, as an invasive procedure, is rarely performed in children with chronic viral hepatitis, and it is not possible to use it as a part of clinical management. Second, we did not perform longitudinal observations but rather a cross-sectional study at the moment the patients were eligible for treatment with DAAs. Thus, it is possible that in some patients, significant fibrosis can develop at an even younger age.

In conclusion, a significant proportion of children who qualify for DAA treatment due to HCV present with significant liver stiffness in TE evaluation. The main predictors of significant fibrosis include age and duration of infection >10 years. There is some agreement between the results of biomarker (APRI and FIB-4) and TE evaluation, but with the assumption of lower cutoff thresholds indicating significant fibrosis/cirrhosis than previously validated in adults.
